# Elastin-specific MR probe for visualization and evaluation of an interleukin-1β targeted therapy for atherosclerosis

**DOI:** 10.1038/s41598-024-71716-5

**Published:** 2024-09-04

**Authors:** Dilyana Branimirova Mangarova, Carolin Reimann, Jan Ole Kaufmann, Jana Möckel, Avan Kader, Lisa Christine Adams, Antje Ludwig, David Onthank, Simon Robinson, Uwe Karst, Rebecca Helmer, Rene Botnar, Bernd Hamm, Marcus Richard Makowski, Julia Brangsch

**Affiliations:** 1 Department of Radiology, Charité – Universitätsmedizin Berlin, Corporate Member of Freie Universität Berlin, Humboldt-Universität zu Berlin, and Berlin Institute of Health, Charitéplatz 1, 10117 Berlin, Germany; 2 Division 1.5 Protein Analysis, Federal Institute for Materials Research and Testing (BAM), Richard-Willstätter-Str. 11, 12489 Berlin, Germany; 3 Department of Diagnostic and Interventional Radiology, Technical University of Munich, Ismaninger Str. 22, 81675 Munich, Germany; 4 Department of Cardiology and Angiology, Charité - Universitätsmedizin Berlin, Corporate Member of Freie Universität Berlin, Humboldt-Universität zu Berlin and Berlin Institute of Health, Berlin, Germany; 5 DZHK (German Center for Cardiovascular Research), Partner Site, Berlin, Germany; 6 Lantheus Medical Imaging, 331 Treble Cove Road, North Billerica, MA United States of America; 7 Institute of Inorganic and Analytical Chemistry, Westfälische Wilhelms-Universität Münster, Corrensstr. 48, 48149 Münster, Germany; 8 School of Biomedical Engineering and Imaging Sciences, King’s College London, St Thomas’ Hospital Westminster Bridge Road, London, SE1 7EH United Kingdom; 9 Wellcome Trust/EPSRC Centre for Medical Engineering, King’s College London, London, United Kingdom; 10 BHF Centre of Excellence, King’s College London, Denmark Hill Campus, 125 Coldharbour Lane, London, SE5 9NU United Kingdom; 11 Escuela de Ingeniería, Pontificia Universidad Católica de Chile, Santiago, Chile

**Keywords:** Atherosclerosis, Molecular imaging, Elastin, Plaque, Interleukin-1beta, Molecular biology, Biomarkers, Molecular medicine

## Abstract

Atherosclerosis is a chronic inflammatory condition of the arteries and represents the primary cause of various cardiovascular diseases. Despite ongoing progress, finding effective anti-inflammatory therapeutic strategies for atherosclerosis remains a challenge. Here, we assessed the potential of molecular magnetic resonance imaging (MRI) to visualize the effects of 01BSUR, an anti-interleukin-1β monoclonal antibody, for treating atherosclerosis in a murine model. Male apolipoprotein E-deficient mice were divided into a therapy group (01BSUR, 2 × 0.3 mg/kg subcutaneously, n = 10) and control group (no treatment, n = 10) and received a high-fat diet for eight weeks. The plaque burden was assessed using an elastin-targeted gadolinium-based contrast probe (0.2 mmol/kg intravenously) on a 3 T MRI scanner. T1-weighted imaging showed a significantly lower contrast-to-noise (CNR) ratio in the 01BSUR group (pre: 3.93042664; post: 8.4007067) compared to the control group (pre: 3.70679168; post: 13.2982156) following administration of the elastin-specific MRI probe (*p* < 0.05). Histological examinations demonstrated a significant reduction in plaque size (*p* < 0.05) and a significant decrease in plaque elastin content (*p* < 0.05) in the treatment group compared to control animals. This study demonstrated that 01BSUR hinders the progression of atherosclerosis in a mouse model. Using an elastin-targeted MRI probe, we could quantify these therapeutic effects in MRI.

## Introduction

The majority of lethal cardiovascular diseases worldwide occur as a result of atherosclerosis, a maladaptive, non-resolving chronic inflammatory disease that occurs at sites of blood flow disturbance^1^. While the trigger for atherosclerosis is not yet fully understood, the so called “bad” cholesterol consisting of low-density lipoproteins (LDL) and other triglyceride-rich lipoproteins, is a key factor for the disease development^2^. Furthermore, macrophages devour the oxidized LDL particles and transform into foam cells, which marks the beginning of the fatty streaks formation^3,4^. Additionally, the resulting cholesterol crystals co-activate the NACHT, LRR, and PYD domains-containing protein 3 (NLRP3)-inflammasome, a multi-protein complex involved in the activation of interleukin-1β (IL-1β)^5^. IL-1β is one of the main cytokines in the inflammatory mechanisms of atherosclerosis, activating and mediating the humoral immune response^6^. Thereby, it does not only activate secondary inflammatory cytokines, like IL-6, but is also responsible for the phenotype transition of vascular smooth muscle cells, responsible for a further increase in IL-1β expression^7^.

By neutralizing IL-1β in the Canakinumab Anti-inflammatory Thrombosis Study (CANTOS) with the monoclonal Canakinumab antibody, a reduction in recurrent cardiovascular events was observed in patients^8,9^. Canakinumab binds to IL-1β with high specificity, thus reducing the risk of cross-reactions to other cytokines and leads to the formation of antigen–antibody complexes^10^. These findings were also validated in a preclinical model of abdominal aortic aneurysm (AAA)^11^, using the mouse-specific monoclonal antibody 01BSUR which binds to IL-1β and led to a decrease in aortic dilatation and preservation of the extracellular matrix (ECM). Regarding atherosclerosis, mouse studies from recent years demonstrated promising results, i.e. plaque remodeling towards a more stable phenotype^12^ and decreased vascular inflammation as a result of anti-inflammatory therapy^5^.

Disturbances in the ECM homeostasis are critically important for plaque stability, migration of inflammatory cells and the retention of lipoproteins, all of which contribute to the progression of atherosclerosis^13^. Elastin is one of the main ECM components in the vascular wall, responsible for its elasticity and structural integrity^14^. In the context of atherosclerosis, disrupted elastogenesis^15^, elastin fragmentation^16^ and degradation^17^ promote plaque development. Therefore, characterizing the elastin content of atherosclerotic plaques might offer important insights into plaque composition and vulnerability^18^. Recently, a low-molecular weight elastin-targeted probe was introduced for molecular magnetic resonance imaging (MRI)^19^. Various studies demonstrated the successful characterization of plaque burden^20^ and stability^21^ in preclinical models of atherosclerosis using elastin-enhanced molecular MRI.

It has been demonstrated previously that IL-1β upregulates elastin gene expression^22^. We hypothesized that 01BSUR treatment alleviates atherosclerosis and its therapeutic effects can be observed on a molecular level using an elastin-specific MR probe.

## Results

### Molecular MR imaging for the in vivo assessment of the effects of IL-1β inhibitor 01BSUR treatment

**Fig. 1:**
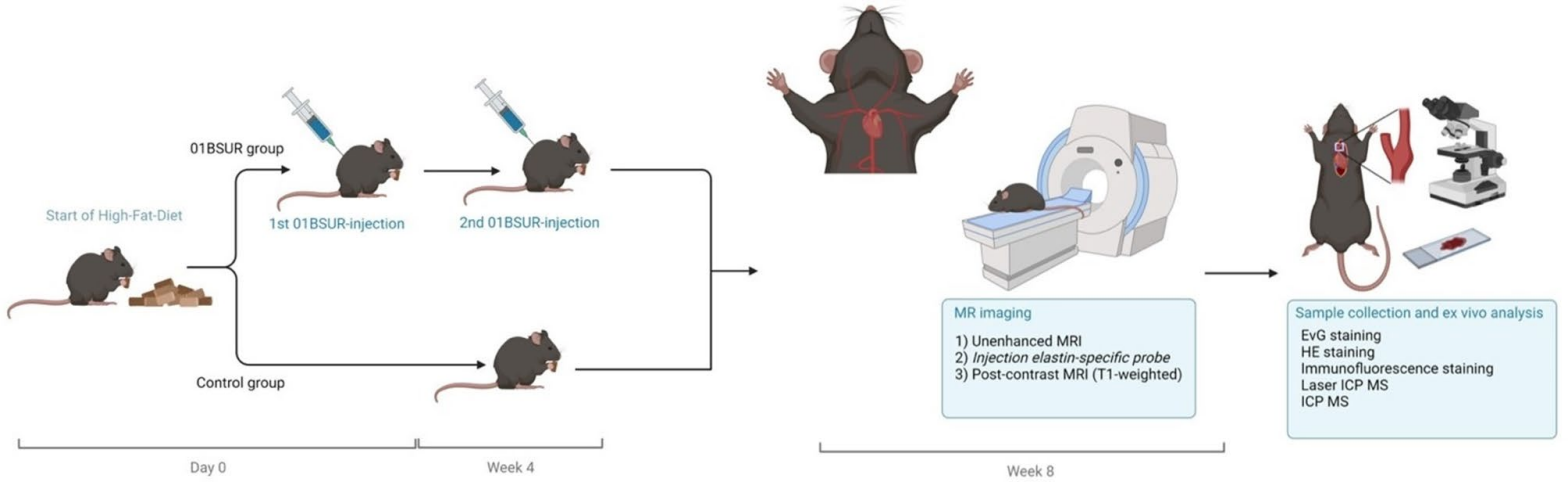
Study design. Two groups of male apolipoprotein E-knockout mice received a high-fat diet. The 01BSUR treatment group (n = 10) received a first subcutaneous injection of the anti-interleukin-1β antibody 01BSUR at the start of the high-fat diet and a second injection 4 weeks later. The control group (n = 10) received no therapy. MR imaging was performed 8 weeks after the start of the high-fat diet. Following the imaging session, animals were sacrificed, and arterial tissue was excised for ex vivo analysis. Created with BioRender.com.

**Fig. 2:**
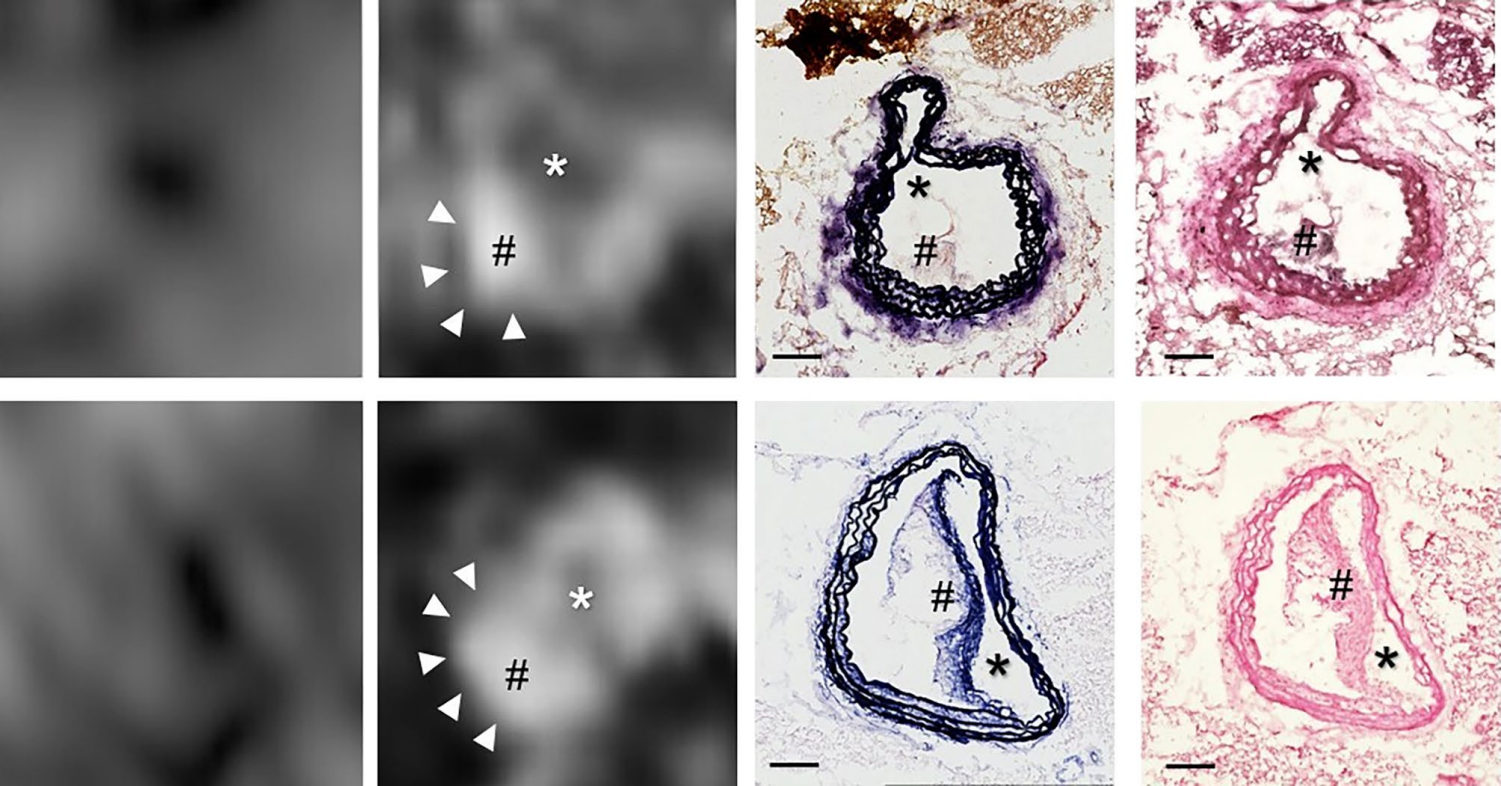
Molecular MRI using an elastin-specific probe for visualisation of atherosclerotic plaque burden. Unenhanced T1-weighted MR images showed no relevant signal of the atherosclerotic plaque area. After the intravenous injection of the elastin-specific probe, a clear signal enhancement (with arrows) was observed in the vessel wall corresponding to the atherosclerotic plaque area on T1-weighted images. Ex vivo histological staining confirmed atherosclerotic plaque formation including the expression of elastic fibers within the plaque area. *MRI* magnetic resonance imaging; *EvG* Elastica van Gieson; *HE* hematoxylin–eosin staining; *indicates the arterial lumen; #indicates the plaque area; Scale bars represent 100 µm.

Apolipoprotein E-deficient (ApoE^−/−^) mice in the control group (n = 10), receiving no treatment with the IL-1β inhibitor 01BSUR, developed visible atherosclerotic plaques in the aortic arch, thoracic aorta, brachiocephalic and carotid arteries after 8 weeks of high-fat diet (HFD) (Fig. 1). Using the elastin-specific probe, elastic fibers within the atherosclerotic plaque could be visualized in vivo. In contrast, the treatment group (n = 10) which received two subcutaneous injections of 01BSUR and 8 weeks of HFD, showed a marked reduction in the development of atherosclerotic plaques compared to the control group, as seen in elastin-enhanced molecular MRI (Fig. 2).

### T1-weighted MR imaging for the assessment of the elastin-specific probe

**Fig. 3:**
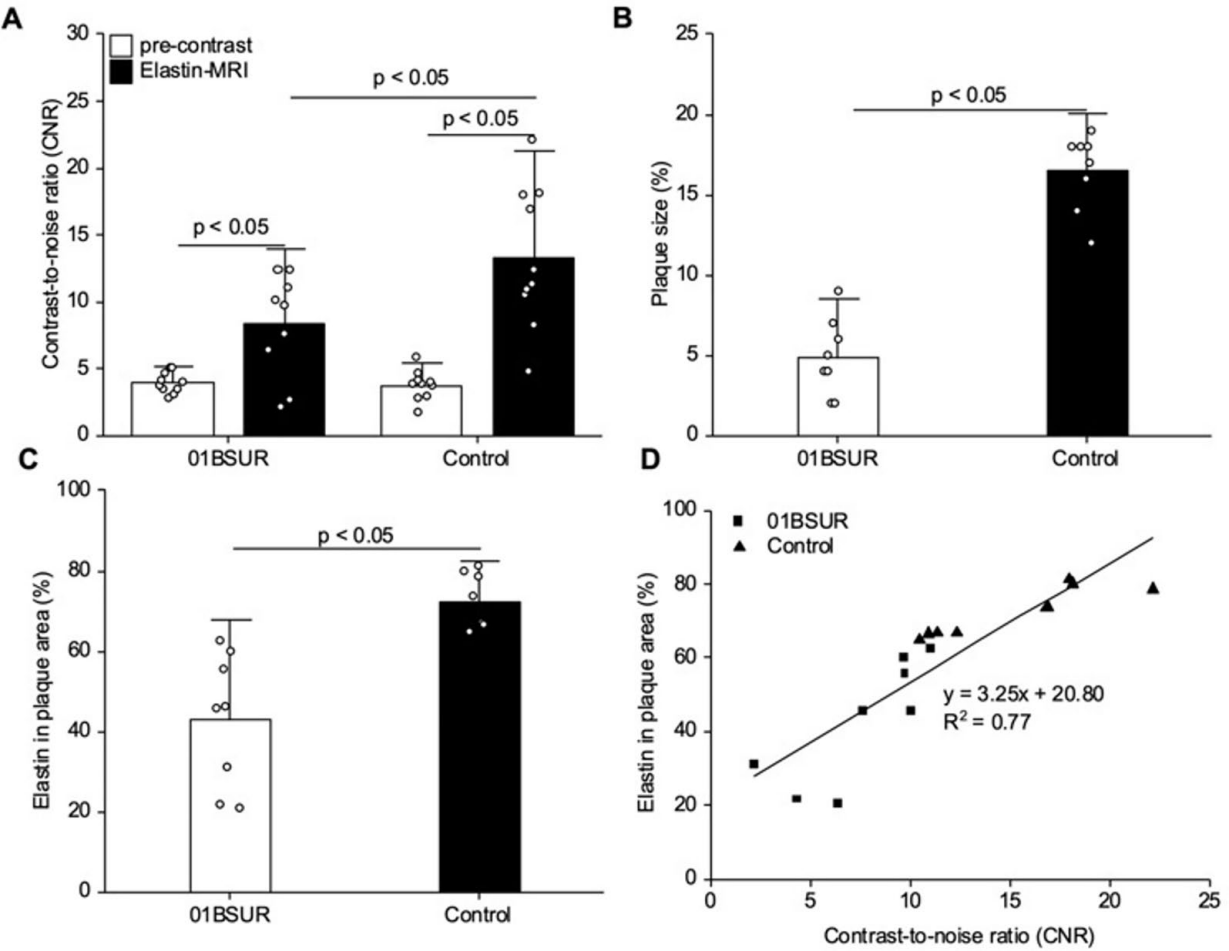
Characterization of the plaque-burden by molecular MRI and ex vivo histology in response to anti-inflammatory therapy. (**A**) A significant increase of contrast-to-noise-ratio was observed after administration of the elastin-specific probe 8 weeks after start of the high-fat diet in both groups (*p* < 0.05). The control group showed a significantly stronger enhancement compared to the 01BSUR group. (**B**, **C**) Mice treated two times with the anti-interleukin-1β antibody 01BSUR showed a significant lower relative plaque size (*p* < 0.05) as well as lower elastin fiber content within the atherosclerotic plaque area (*p* < 0.05) compared to mice of the control group. (**D**) In vivo MRI measurements of the contrast-to-noise area were in good correlation with ex vivo histological analyses (y = 3.25x + 20.80, R^2^ = 0.77). All data are presented as mean values ± SD.

In the area of the aortic arch, brachiocephalic and carotid arteries, a low contrast-to-noise ratio (CNR) was shown in both the treatment and control group prior to the intravenous injection of the gadolinium-based elastin-specific probe. T1-weighted imaging after the administration of the elastin-specific probe showed a significant increase (*p* < 0.05) in CNR in both groups: Mice of the 01BSUR treatment group showed a twofold increase in CNR in the area of the brachiocephalic vessel compared to the unenhanced scan (pre: 3.93042664; post: 8.4007067) whereas in the control group, the MR signal was over 3.5 times higher than prior to the administration of the elastin-specific probe (pre: 3.70679168; post: 13.2982156; Fig. 3A).

### Histological analyses

#### Analysis of plaque size and elastin amount by Elastica van Giesson (EvG) staining

To evaluate the effect of the 01BSUR treatment on atherosclerotic plaque formation, Elastica van Giesson (EvG) staining was performed to assess the plaque size and the amount of elastic fibers in the plaque area. Mice of the 01BSUR treatment group showed a significant reduction in plaque formation and relative size (*p* < 0.05) compared to mice of the control group (01BSUR group: 4.9%, control group: 16.5%, Fig. 3B). Moreover, EvG staining demonstrated a significant decrease in the amount of elastin in the plaque area of the 01BSUR treatment group (42.86% ± 16.66%) compared to the control group (72.30% ± 6.87%, p < 0.05, Fig. 3C).

Histological measurements of elastin content were in good correlation with the in vivo MR measurements of signal enhancement after administration of the elastin-specific probe (y = 3.25x − 20.80 R^2^ = 0.77; Fig. 3D).

#### Analysis of inflammation of the atherosclerotic arterial wall by immunofluorescence

**Fig. 4:**
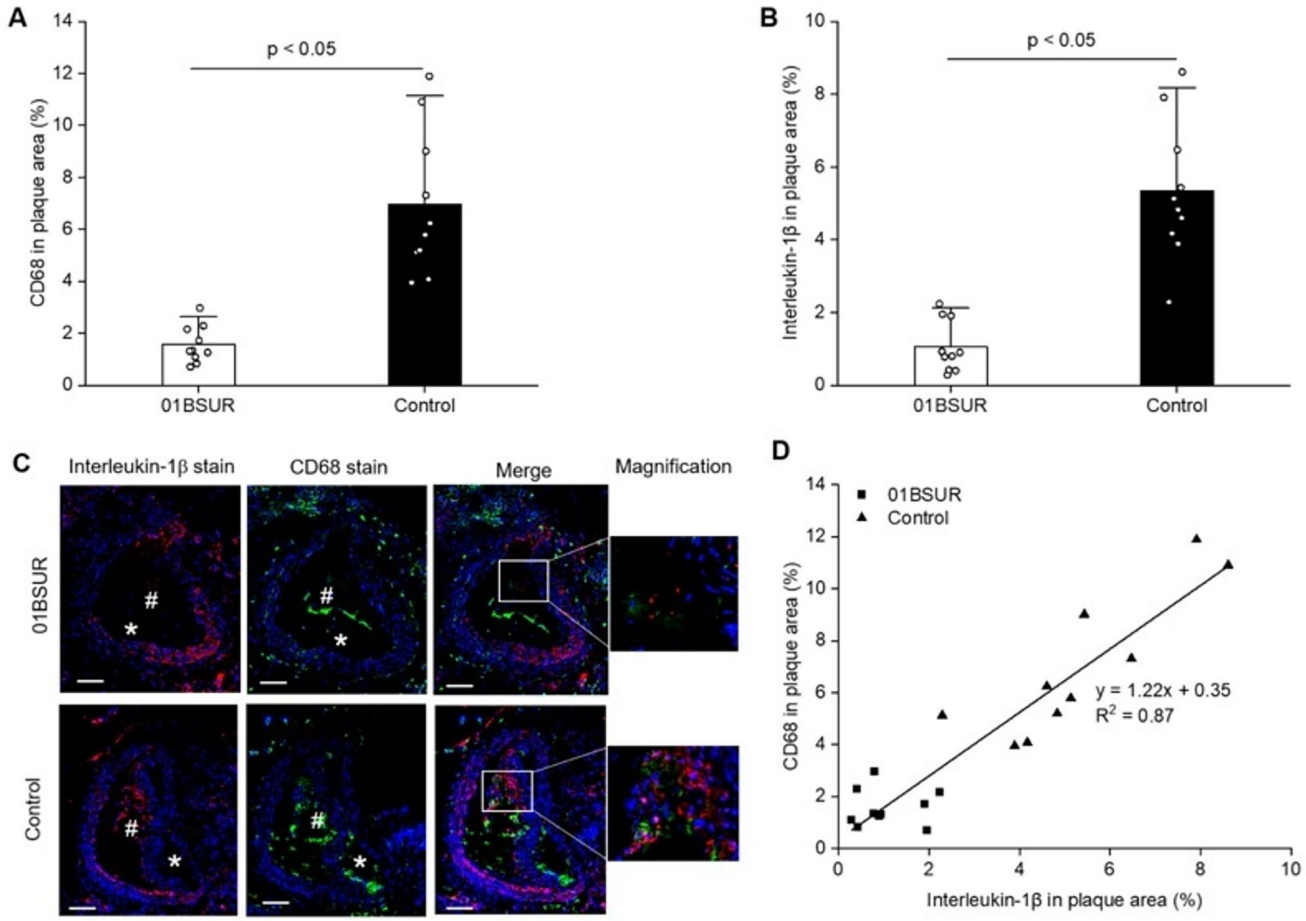
Ex vivo histological analyses of inflammatory markers. (**A**, **B**) Mice treated two times with 01BSUR showed a significant lower expression of CD68 + macrophages (*p* < 0.05) and interleukin-1β (*p* < 0.05) in atherosclerotic plaque area after 8 weeks of high-fat diet compared to mice of the control group. (**C**) Immunofluorescence analyses: Macrophages (CD68 +) are shown in green, interleukin-1β in red and cell nuclei (DAPI) in blue. The stacked image shows a co-localization of interleukin-1β with macrophages. (**D**) The amount of CD68 + macrophages and IL-1β-expression within the atherosclerotic plaque area were in good correlation (y = 1.22x + 0.35; R^2^ = 0.87). *indicates the arterial lumen; #indicates the plaque area; Scale bars represent 100 µm.

For evaluation of the inflammatory processes within the atherosclerotic plaque, different immunofluorescence (IF) stainings were performed. The presence of CD68 + macrophages within the plaque area was evaluated and demonstrated a fourfold decrease (Fig. 4A) in the 01BSUR treatment group (1.568%) compared to the control mice (6.951%), indicating a significant (*p* < 0.5) reduction of inflammation within the atherosclerotic arterial wall by 01BSUR treatment. Importantly, IF staining against IL-1β demonstrated five times lower interleukin-1β amount within the plaque area in 01BSUR treated mice compared to the non-treated control group (Fig. 4B). Furthermore, a clear colocalization of areas positive for IL-1β and CD68 was observed (Fig. 4C). Additionally, a strong correlation between the absolute amount of interleukin-1β and CD68 within the plaque area was measured (y = 1.22x + 0.35, R2 = 0.87, Fig. 4D).

#### Spatial distribution of the gadolinium-based elastin-specific probe by laser-ablation inductively coupled plasma mass spectrometry (LA-ICP-MS)

**Fig. 5:**
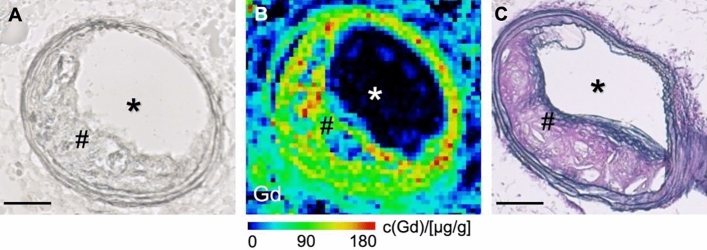
Laser-ablation inductively coupled plasma mass spectrometry (LA-ICP-MS) for spatial localization of gadolinium in the atherosclerotic plaque area. (**A**) Unenhanced light microscopy image of an animal (n = 1) from the control group. (**B**) Corresponding image of the LA-ICP-MS visualizing the gadolinium-based elastin-specific probe within atherosclerotic plaque by the gadolinium signal. (**C**) Elastica van Gieson staining revealed a clear colocalization of gadolinium-accumulation with elastic fibers in the atherosclerotic plaque. *indicates the arterial lumen; #indicates the plaque area; Scale bars represent 100 µm.

For visualization and spatial localization of the gadolinium-based elastin-specific probe in the atherosclerotic arterial wall, LA-ICP-MS was performed (n = 1). On histological sections of the brachiocephalic artery a strong co-localization of targeted gadolinium with elastic fibers was demonstrated (Fig. 5).

## Discussion

In the current study, we demonstrated the feasibility of molecular MRI using an elastin-specific probe to visualize and evaluate an IL-1β targeted anti-inflammatory therapy against atherosclerosis in a murine model.

### The role of interleukin-1β in the development of cardiovascular disease

Through binding and neutralizing the cytokine IL-1β, the antibody 01BSUR decreases the pro-inflammatory and tissue-degrading activities of interleukin which results in a marked reduction of the development and progression of a variety of acute and chronic inflammatory diseases. In the case of atherosclerosis, IL-1β promotes the exacerbation of established atherogenesis^23,24^. In the present study, ex vivo analyses revealed a significantly lower IL-1β expression within the atherosclerotic lesion in 01BSUR-treated mice compared to the non-treated control group. These findings underline the critical pro-inflammatory role of interleukin-1β as one of the key mediators of inflammation during the formation and progression of atherosclerotic plaques. Produced by intrinsic vascular wall cells and lesional leukocytes, IL-1β induces procoagulant activity and promotes especially the adhesion of monocytes and leukocytes to vascular endothelial cells, as well as the growth of vascular smooth muscle cells, thus initiating plaque development, and influencing plaque stability^1,8,25^. Our results are in line with Kirii et al.^26^, demonstrating that a genetic deletion of IL-1β in ApoE^−/−^ mice reduces atherosclerotic lesions by 33%, whereas the deficiency of IL-1 receptor antagonist (IL-1Ra) significantly increased the atherosclerotic lesions formed^27^. Furthermore, chronic treatment with IL-1β in vivo caused coronary intimal lesions and vasospastic responses in porcine coronary arteries^28^.

Collectively, these studies suggest that interfering with IL-1β signaling in the vascular wall, adopting strategies such as upregulation of the activity of IL-1Ra, downregulation of IL-1 production in the vascular wall or effecting IL-1 receptor signaling, can potentially be used as a therapeutic target in atherosclerosis^25^. With this study, we aimed to visualize those effects using elastin-enhanced molecular MRI.

### Anti-inflammatory therapies in atherosclerosis

In recent years, several different inflammatory pathways have been identified in the initiation and progression of atherosclerosis. Following the CANTOS study, in which the effects of IL-1β antibody were evaluated in patients after myocardial infarction^8^, several studies have investigated the use of 01BSUR against atherosclerosis in preclinical models and produced conflicting results. Here, we found a significant reduction in plaque size, elastin and macrophage content in the treatment group compared to the controls. In a study by Dragoljevic et al., 01BSUR treatment led to a reduction in macrophage and lipid content, yet an increase in collagen content, speculating that those changes lead to plaque stabilization^12^. Hettwer et al. also reported positive effects of 01BSUR, reflected in reduced inflammatory markers, total plaque size and smaller necrotic cores^5^. On the contrary, Gomez et al. argue that endogenous IL-1β has atheroprotective effects, since treatment with 01BSUR led to a decreased smooth muscle cell and collagen content and increased macrophage content in the fibrous cap, while no reduction of plaque size was seen^29^. It must be pointed out that the abovementioned studies used different mouse strains, atherosclerosis induction techniques and treatment regimens, which might explain the contradictory findings.

A large-scale clinical trial demonstrated that a low dose of colchicine significantly lowers the risk of ischemic cardiovascular events following a recent myocardial infarction^30^. Colchicine inhibits tubulin polymerization^30,31^ and limits the NLRP3 inflammasome activity, consequently suppressing IL-1β activity^32^. In a rabbit model of atherosclerosis, colchicine treatment resulted in plaque stabilization by reducing the plaque burden and inflammatory activity without a marked macrophage reduction^33^. This is partially in line with the results from our mouse model under 01BSUR therapy, where we found a reduction in plaque size and a statistically significant reduction of CD68 + cells in the vascular wall.

Another common target for cardiovascular trials is IL-6. Tocilizumab, an IL-6 antibody has been shown to improve the clinical outcome for patients with myocardial infarction by rapidly modulating neutrophile function^34,35^. Other drug targets such as methotrexate, an antimetabolite and immunosuppressant, did not lead to a significant reduction of pro-atherosclerotic markers, including IL-1β^36^.

### Effects of IL-1β on elastin metabolism in cardiovascular disease

Previous studies have demonstrated a relationship between IL-1β and elastin metabolism in the development of cardiovascular disease. In animal models of aneurysms and aortic dissections, disease induction led to elevated IL-1β tissue levels, and concurrently, breakage and reduction in elastic fiber content^37,38^. Conversely, treatment with an IL-1β antibody or genetic depletion of IL-1β in these animal models led to preservation of elastin fiber levels and morphology^37,39^. Regarding atherosclerosis, ApoE^−/−^ IL-1Ra^−/−^ mice showed more pronounced signs of arterial inflammation and destruction of the elastic laminae during plaque development compared to ApoE^−/−^ mice^40^. Taken together, these results suggest that with progressing atherosclerosis, an increase in local IL-1β and decrease in elastin occurs. In contradiction with those results, we found that 01BSUR therapy resulted in lower elastin content in atherosclerotic plaques compared to non-treated animals. A possible explanation for this contradiction is the dynamic interplay between elastolysis and elastogenesis. It has been demonstrated that IL-1β upregulates elastin gene expression^22^ and ineffective elastin synthesis takes place during atherosclerosis progression^15^. In turn, this can lead to an increase in total elastin content^20,21^ in advanced disease stages. The resulting immature elastin, in combination with destructed elastic fiber fragments feeds into a vicious cycle of pathological elastin remodeling^41^.

### Therapy monitoring using molecular elastin-specific MR imaging

So far, the elastin-specific probe used in this study has been successfully implemented for therapy monitoring in several preclinical models. Here, we showed its efficacy for non-invasive detection of the therapeutic effects of a murine IL-1β antibody on a molecular level. These results are in line with data from an abdominal aortic aneurysm (AAA) model, where 01BSUR therapy prevented extensive aortic dilatation and elastin destruction. These findings were confirmed with elastin-enhanced MRI^11^. Similarly, Makowski et al. found a decrease of CNR and relative atheroma size following statin therapy in an ApoE^−/−^ mouse model of progressive atherosclerosis^20^. Beyond cardiovascular disease, elastin-enhanced MR imaging allowed for longitudinal monitoring of the therapeutic effects of an anti-fibrotic drug^42^ in kidney fibrosis. Moreover, the beneficial effects of non-invasive elastin imaging go beyond pharmacological therapies. Following thermal ablation of liver cancer, it is possible to quantitively asses the ablation-induced ECM remodeling in the periablational rim^43^.

### Translational potential and limitations

The use of a 3 T clinical MRI scanner instead of a high-field preclinical device in this study represents a key factor for future translation into patients. Moreover, the drug dosages and administration routes for therapy and imaging were based on the clinical guidelines for Canakinumab^8^ and Gd-based contrast agents, respectively^20^. The size, molecular composition and blood half-life of the elastin-specific probe are comparable to that of clinically approved MRI contrast agents^20^.

While we yielded some promising results, our study also has several limitations. First, there are some important dissimilarities regarding atherosclerosis development between ApoE^−/−^ mice and humans. ApoE^−/−^ mice develop accelerated atherosclerosis, with initial lesions being visible as early as 6 weeks of age^44^. In our study, the first dose of 01BSUR was administered at 8 weeks of age, shortly after initial plaque formation, in parallel with the high fat diet start. In the future, it would be beneficial to test the efficacy of 01BSUR on late-stage atherosclerotic plaques. Atheroma development in humans starts later in life and the progression spans over several decades^45^. Additionally, the predominant type of plasma cholesterol in hyperlipidemic ApoE^−/−^ mice is very low-density lipoproteins (VLDL), compared to low-density lipoproteins (LDL) in humans^46,47^. Future studies focusing on plaque composition on the molecular level and associated risk factors in both animal models and human cohorts are needed to establish the significance of regional atheroma morphology for cardiovascular events.

Second, only male mice were examined in this study. Previous literature reports differences in atheroma development^48^ and response to environmental triggers^49^ in male and female atherosclerotic ApoE^−/−^ mice. Adding a female subgroup in future experiments would allow us to study sex-specific therapy response differences.

To conclude, we demonstrated that 01BSUR hinders the progression of atherosclerosis by reducing plaque size, inflammatory markers, and elastin content. Using an elastin-specific molecular probe, we could non-invasively quantify the therapeutic success of an anti-IL-1β by signal intensity measurements. In the future, molecular MRI techniques may support the diagnosis and treatment of atherosclerosis by characterization of plaque composition and non-invasive monitoring of pharmacological therapies in patients.

## Methods

### Animal experiments

All animal experiments were performed with respect to the guidelines and regulations of the Federation of Laboratory Animal Science Associations (FELASA) and the local Guidelines and Provisions for Implementation of the Animal Welfare Act and were approved by the Landesamt für Gesundheit und Soziales (LaGeSo) Berlin. The study is reported in accordance with the ARRIVE guidelines for reporting animal research when applicable. Randomization was performed when allocating the animals to each group. Blinding was not possible during the in vivo experiments and was not performed during data analysis.

The mice used in this study were obtained from the Research Institute of Experimental Medicine at the Charité–Universitätsmedizin Berlin. Maintenance of the animals included barrier conditions, a dark/light cycle of 12 h, an ambient temperature of 20 °C, and a humidity of 45%. Two groups of apolipoprotein E-knockout (B6.129P2-ApoE^tm1Unc^/J) mice (male, 8-week-old, n = 10 per group) were used. Both groups were fed a HFD containing 22% fat (C 1090-45; Altromin Spezialfutter GmbH, Lage, Germany) for 8 weeks, starting at the age of 8 weeks. The diet as well as water were provided ad libitum. To evaluate the therapeutic effect of the anti-IL-1β antibody 01BSUR on atherosclerotic plaque formation, one group (01BSUR treatment group, n = 10) received subcutaneous injections of 01BSUR (Novartis, Basel, Switzerland, 0.3 mg/kg) twice: the first injection at the start of the HFD and the second injection 4 weeks after the start of the HFD. The second group of mice (n = 10), received no therapy, serving as the control group (Fig. 1).

MRI was performed 8 weeks after the start of the HFD. First, mice were anaesthetized using a combination of Medetomidine (500 µg/kg), Fentanyl (50 µg/kg), and Midazolam (5 mg/kg) which were administered intraperitoneally. Second, unenhanced MR imaging was performed, followed by the intravenous injection of the elastin-specific probe (Lantheus Medical Imaging, North Billerica, Massachusetts, USA; 0.2 mmol/kg) and further imaging.

Following the imaging session, animals were sacrificed by cervical dislocation and exsanguinated by arterial perfusion of saline. The brachiocephalic and carotid arteries, aortic arch and thoracic aorta were excised for ex vivo analysis.

### Interleukin-1β inhibitor 01BSUR

In this study, mice in the treatment group (n = 10) received the IL-1β inhibitor 01BSUR (Novartis, Basel, Switzerland) two times: the first injection (s.c.) at the start of the HFD and the second s.c. injection 4 weeks after the start of the HFD. A dosage of 0.3 mg per mouse per administration was used (0.6 mg per mouse in total), equivalent to the clinically approved dose of Canakinumab used in the CANTOS study^8^. 01BSUR represents an IgG2a/k monoclonal mouse antibody that binds with high specificity to IL-1β with a dissociation constant of (K_D_) = 302 pM and a half-life of 14 days^50^. Due to its anti-inflammatory effects, the antibody has already been evaluated in various other models of diseases, including aortic aneurysms^50^ as well as aortic calcification^51^ and collagen-induced arthritis^52^.

### Gadolinium-based elastin-specific contrast probe

For visualization and characterization of the arterial wall, we used an elastin-specific probe (Lantheus Medical Imaging, North Billerica, Massachusetts, USA) based on gadolinium). This probe has already been studied for the evaluation of atherosclerosis progression^53^ and AAAs in mice^54–56^. With a molecular weight of 856 g/mol, a short blood half-life and a predominant clearance by the renal system, the probe is highly comparable to contrast agents already used in clinical practice^20^. Following intravenous injection, the highest uptake within the aorta was shown after 30 to 45 min (13.2 ± 2.3% ID g^−1^)^20^.

### In vivo MR imaging

Mice in the treatment (n = 10) and control group (n = 10) were imaged after 8 weeks of HFD. For MR imaging, a clinical 3 T MR Scanner (Biograph, Siemens Healthcare Solutions, Erlangen, Germany) in combination with a clinical single loop coil (47 mm, Siemens Healthcare Solutions, Erlangen, Germany) was used. The anesthetized mice received a venous access via the tail vein for administration of the contrast probe and were then placed in a prone position on the coil. During the image session, the body temperature (37 °C) of the animals was constantly monitored with an MR-compatible heating system (Model 1025, SA Instruments Inc, Stony Brook, NY).

MR imaging was performed as previously described^53^:

For an anatomical overview and localization of the thoracic aorta, carotid and brachiocephalic arteries, low-resolution three-dimensional gradient echo scout scan was performed in sagittal, coronal and transverse orientation using the following parameters: field-of-view (FOV) = 280 mm, matrix = 320, slice thickness = 3 mm, TR/TE = 7.7/3.7 ms, flip angle = 20° and slices = 10. The scout scan was followed by a two-dimensional (2D) time-of-flight (TOF) scan in transverse orientation for visualization of the aortic arch and the brachiocephalic artery. Imaging parameters included: FOV = 200 mm, matrix = 960, in plane spatial resolution = 0.2 × 0.2 mm, slice thickness = 500 µm, TR/TE = 35/4.5 ms, flip angle = 90° and slices = 26. From the TOF dataset, a maximum intensity projection (MIP) was generated for display of an arterial angiogram of the aortic arch, the brachiocephalic and carotid arteries to plan the subsequent contrast-enhanced sequences. The inversion recovery scan to visualize the G-based contrast agent was preceded by a 2D Look-Locker sequence planned perpendicular to the ascending aorta, which was used to determine the optimal inversion time (TI) for blood signal nulling. Imaging parameters of the Lock Locker sequence included: FOV = 300 mm, matrix = 750, in plane spatial resolution = 0.4 × 0.4 mm, slice thickness = 1.5 mm, TR between subsequent IR pulses = 1000 ms, and flip angle = 15°. Imaging parameters of the high-resolution 3D inversion recovery gradient echo late Gd enhancement (LGE) sequence scan employed for visualization of G-based molecular probe were: FOV = 57 mm, matrix = 416, in plane spatial resolution = 0.137 × 0.137 mm, slice thickness = 370 µm, slices = 56, TR/TE = 12.1/5.7 ms, TR between subsequent IR pulses = 1000 ms, and flip angle = 30°.

### Assessment of magnetic resonance imaging signal

Quantification of the MRI signal and morphometric measurements were conducted on high–resolution MR images as previously described^54^.

Using OsiriX (version 7.1, OsiriX foundation), Regions Of Interest (ROIs) were co–localized with the atherosclerotic plaque (highest signal within the arterial wall) and defined as areas of enhancement. The CNR was then calculated: CNR = (Combined vessel wall and atherosclerotic-plaque-signal − Blood signal)/Noise. The noise was defined as the standard deviation in pixel intensity from a ROI placed in the background air anterior to the brachiocephalic artery.

### Histological analysis of the atherosclerotic plaques

For histological analysis, arterial tissue, including the brachiocephalic and carotid arteries, aortic arch and thoracic aorta, was frozen at − 20 °C following excision. Then tissues were embedded in optimal cutting temperature (OCT) compound (Sakura Finetek, Breisgau, Germany) and subsequently cut into 10 μm cryosections. Samples were mounted on SuperFrost Plus adhesion slides (Thermo Fisher Scientific, Waltham, USA) and stained using Miller’s Elastica van Gieson stain (EvG) and Hematoxylin and Eosin (HE).

For further morphometric analysis and elastin quantification, slides were examined and photographed using a digital light microscope (BZ-X800, Keyence, Osaka, Japan) and corresponding Keyence BZ-X800 Analyzer software (Keyence, Osaka, Japan). The %EvG stain area per vessel cross section was determined as previously described^54,57^:

First, a reference standard was set by marking the color profile of the normal elastic laminae in the medial wall. Any structure with this specific color profile within the adventitia was automatically segmented and the resulting areas were documented. We then divided the segmented area by the overall vessel surface and thus determined the %EvG stain per vessel cross-section.

### Immunofluorescence analysis

For the visualization and subsequent quantification of IL-1β and CD68 + macrophages within the arterial wall, immunofluorescence (IF) staining was performed. Cryosections were obtained as described above. First, antibodies were diluted in Dako REAL Antibody Diluent (Dako, Glostrup, Denmark). Second, sections were incubated with a polyclonal IL-1β antibody (rabbit anti-mouse IL-1 beta, 1:100 dilution, Bio-Rad, Hercules, USA) or a monoclonal CD68 antibody (rat anti-mouse MCA1957, 1:100 dilution, BioRad, Hercules, USA) overnight at 4 °C. After a wash using PBST (0.05% Tween20, pH 7.4), slides were incubated with polyclonal secondary antibody labeled with AlexaFluor 568 (donkey anti-rabbit IgG, Thermo Fisher Scientific, Germany, 1:200 dilution or goat anti-rat A11077, 1:200 dilution, Invitrogen, Waltham, USA) for 1 h at room temperature. Subsequent counterstaining and mounting were performed with Roti^®^-Mount FluorCare (Carl Roth, Germany).

For interleukin and CD68 + quantification, slides were examined and photographed using a digital light microscope (BZ-X800, Keyence, Osaka, Japan). Subsequent morphometric analysis was performed using Keyence BZ-X800 Analyzer software (Keyence, Osaka, Japan).

### Element-specific bioimaging using laser-ablation inductively coupled plasma mass spectrometry (LA-ICP-MS)

LA-ICP-MS was performed as described previously^54^. Arterial tissue was cut into 10 µm cryosections at − 20 °C and mounted on SuperFrost Plus adhesion slides (Thermo Fisher Scientific, Waltham, MA, USA). The LA-ICP-MS analysis was performed using an LSX 213 G2 + laser system (CETAC Technologies, Omaha, USA) which was equipped with a two-volume HelEx II cell connected via Tygon tubing to an ICPMS-2030 (Shimadzu, Kyoto, Japan). With a spot size of 15 µm samples were ablated via line-by-line scan using a scan speed of 45 µm/s and 800 mL/min He as transport gas. The analysis was performed in collision gas mode with He as collision gas, with 50 ms integration time for the analyzed isotopes ^31^P, ^57^Fe, and ^64^Zn and 75 ms integration time for the two Gd isotopes ^158^Gd and ^160^Gd. Matrix-matched standards based on gelatin were used for the quantification of Gd. Nine gelatin standards (10% w/w) including a blank, were spiked with different Gd concentrations (1 to 500 µg/g). Averaged intensities of the scanned lines of the standards were in good linear correlation with a regression coefficient R^2^ = 0.991 within this concentration range. Limit of detection (LOD) and limit of quantification (LOQ), calculated with the 3σ- and 10σ-criteria, were 21 ng/g and 71 ng/g Gd. The quantification and visualization were performed using in-house developed software (WWU Münster, Münster, Germany).

### Statistical analysis

The statistical analysis was performed using Origin 2022 (OriginLab Corporation, Northhampton, USA). Values are specified as mean ± standard deviation. Normal distribution was examined via Shapiro–Wilk test. Statistical comparison of groups was performed by analysis of variance (ANOVA) and Sheffe’s test. An unpaired, two-tailed Student’s *t*-test was applied for the comparison of continuous variables. Linear regression was applied to determine the relationship between in vivo and the ex vivo measurements. A *p*-value < 0.05 was considered to indicate a statistically significant difference.

## Data Availability

The datasets generated during and/or analyzed during the current study are available from the corresponding author on reasonable request.

## References

[1] Hansson, G. K., Libby, P. & Tabas, I. Inflammation and plaque vulnerability. *J. Intern. Med.* **278**, 483–493. 10.1111/joim.12406 (2015). 26260307 10.1111/joim.12406 PMC5082111

[2] Libby, P. The changing landscape of atherosclerosis. *Nature* **592**, 524–533. 10.1038/s41586-021-03392-8 (2021). 33883728 10.1038/s41586-021-03392-8

[3] Falk, E. Pathogenesis of atherosclerosis. *J. Am. Coll. Cardiol.* **47**, C7-12. 10.1016/j.jacc.2005.09.068 (2006). 16631513 10.1016/j.jacc.2005.09.068

[4] Hartley, A., Haskard, D. & Khamis, R. Oxidized LDL and anti-oxidized LDL antibodies in atherosclerosis-novel insights and future directions in diagnosis and therapy<sup/>. *Trends Cardiovasc. Med.* **29**, 22–26. 10.1016/j.tcm.2018.05.010 (2019). 29934015 10.1016/j.tcm.2018.05.010

[5] Hettwer, J. *et al.* Interleukin-1beta suppression dampens inflammatory leucocyte production and uptake in atherosclerosis. *Cardiovasc. Res.* **118**, 2778–2791. 10.1093/cvr/cvab337 (2022). 34718444 10.1093/cvr/cvab337 PMC9586563

[6] Grebe, A., Hoss, F. & Latz, E. NLRP3 inflammasome and the il-1 pathway in atherosclerosis. *Circ. Res.* **122**, 1722–1740. 10.1161/CIRCRESAHA.118.311362 (2018). 29880500 10.1161/CIRCRESAHA.118.311362

[7] Orr, A. W., Hastings, N. E., Blackman, B. R. & Wamhoff, B. R. Complex regulation and function of the inflammatory smooth muscle cell phenotype in atherosclerosis. *J. Vasc. Res.* **47**, 168–180. 10.1159/000250095 (2010). 19851078 10.1159/000250095 PMC2842170

[8] Ridker, P. M. *et al.* Antiinflammatory therapy with canakinumab for atherosclerotic disease. *N. Engl. J. Med.* **377**, 1119–1131. 10.1056/NEJMoa1707914 (2017). 28845751 10.1056/NEJMoa1707914

[9] Ridker, P. M. *et al.* Relationship of C-reactive protein reduction to cardiovascular event reduction following treatment with canakinumab: A secondary analysis from the CANTOS randomised controlled trial. *Lancet* **391**, 319–328. 10.1016/S0140-6736(17)32814-3 (2018). 29146124 10.1016/S0140-6736(17)32814-3

[10] Rondeau, J. M., Ramage, P., Zurini, M. & Gram, H. The molecular mode of action and species specificity of canakinumab, a human monoclonal antibody neutralizing IL-1beta. *MAbs* **7**, 1151–1160. 10.1080/19420862.2015.1081323 (2015). 26284424 10.1080/19420862.2015.1081323 PMC4966334

[11] Brangsch, J. *et al.* Molecular MR-imaging for noninvasive quantification of the anti-inflammatory effect of targeting interleukin-1beta in a mouse model of aortic aneurysm. *Mol. Imaging* **19**, 1536012120961875. 10.1177/1536012120961875 (2020). 33216687 10.1177/1536012120961875 PMC7682246

[12] Dragoljevic, D. *et al.* Inhibition of interleukin-1beta signalling promotes atherosclerotic lesion remodelling in mice with inflammatory arthritis. *Clin. Transl. Immunol.* **9**, e1206. 10.1002/cti2.1206 (2020). 10.1002/cti2.1206 PMC7652637 33204425

[13] Katsuda, S. & Kaji, T. Atherosclerosis and extracellular matrix. *J. Atheroscler. Thromb.* **10**, 267–274. 10.5551/jat.10.267 (2003). 14718743 10.5551/jat.10.267

[14] Lin, C. J., Cocciolone, A. J. & Wagenseil, J. E. Elastin, arterial mechanics, and stenosis. *Am. J. Physiol. Cell Physiol.* **322**, C875–C886. 10.1152/ajpcell.00448.2021 (2022). 35196168 10.1152/ajpcell.00448.2021 PMC9037699

[15] Krettek, A., Sukhova, G. K. & Libby, P. Elastogenesis in human arterial disease: A role for macrophages in disordered elastin synthesis. *Arterioscler. Thromb. Vasc. Biol.* **23**, 582–587. 10.1161/01.ATV.0000064372.78561.A5 (2003). 12615674 10.1161/01.ATV.0000064372.78561.A5

[16] Van der Donckt, C. *et al.* Elastin fragmentation in atherosclerotic mice leads to intraplaque neovascularization, plaque rupture, myocardial infarction, stroke, and sudden death. *Eur. Heart J.* **36**, 1049–1058. 10.1093/eurheartj/ehu041 (2015). 24553721 10.1093/eurheartj/ehu041 PMC4416138

[17] Gayral, S. *et al.* Elastin-derived peptides potentiate atherosclerosis through the immune Neu1-PI3Kgamma pathway. *Cardiovasc. Res.* **102**, 118–127. 10.1093/cvr/cvt336 (2014). 24357053 10.1093/cvr/cvt336

[18] Akima, T. *et al.* Soluble elastin decreases in the progress of atheroma formation in human aorta. *Circ. J.* **73**, 2154–2162. 10.1253/circj.cj-09-0104 (2009). 19755752 10.1253/circj.cj-09-0104

[19] Onthank, D. *et al.* BMS753951: A novel low molecular weight magnetic resonance contrast agent selective for arterial wall imaging. *Circulation* **116**, 411–412 (2007).17606844

[20] Makowski, M. R. *et al.* Assessment of atherosclerotic plaque burden with an elastin-specific magnetic resonance contrast agent. *Nat. Med.* **17**, 383–388. 10.1038/nm.2310 (2011). 21336283 10.1038/nm.2310

[21] Phinikaridou, A. *et al.* Vascular remodeling and plaque vulnerability in a rabbit model of atherosclerosis: Comparison of delayed-enhancement MR imaging with an elastin-specific contrast agent and unenhanced black-blood MR imaging. *Radiology* **271**, 390–399. 10.1148/radiol.13130502 (2014). 24475852 10.1148/radiol.13130502

[22] Mauviel, A. *et al.* Human recombinant interleukin-1 beta up-regulates elastin gene expression in dermal fibroblasts. Evidence for transcriptional regulation in vitro and in vivo. *J. Biol. Chem.* **268**, 6520–6524 (1993). 8454621 10.1016/S0021-9258(18)53281-6

[23] Vromman, A. *et al.* Stage-dependent differential effects of interleukin-1 isoforms on experimental atherosclerosis. *Eur. Heart J.* **40**, 2482–2491. 10.1093/eurheartj/ehz008 (2019). 30698710 10.1093/eurheartj/ehz008 PMC6685323

[24] Dinarello, C. A., Simon, A. & van der Meer, J. W. Treating inflammation by blocking interleukin-1 in a broad spectrum of diseases. *Nat. Rev. Drug Discov.* **11**, 633–652. 10.1038/nrd3800 (2012). 22850787 10.1038/nrd3800 PMC3644509

[25] Kamari, Y. *et al.* Differential role and tissue specificity of interleukin-1alpha gene expression in atherogenesis and lipid metabolism. *Atherosclerosis* **195**, 31–38. 10.1016/j.atherosclerosis.2006.11.026 (2007). 17173923 10.1016/j.atherosclerosis.2006.11.026

[26] Kirii, H. *et al.* Lack of interleukin-1beta decreases the severity of atherosclerosis in ApoE-deficient mice. *Arterioscler. Thromb. Vasc. Biol.* **23**, 656–660. 10.1161/01.ATV.0000064374.15232.C3 (2003). 12615675 10.1161/01.ATV.0000064374.15232.C3

[27] Isoda, K. *et al.* Lack of interleukin-1 receptor antagonist modulates plaque composition in apolipoprotein E-deficient mice. *Arterioscler. Thromb. Vasc. Biol.* **24**, 1068–1073. 10.1161/01.ATV.0000127025.48140.a3 (2004). 15059807 10.1161/01.ATV.0000127025.48140.a3

[28] Shimokawa, H. *et al.* Chronic treatment with interleukin-1 beta induces coronary intimal lesions and vasospastic responses in pigs in vivo. The role of platelet-derived growth factor. *J. Clin. Invest.* **97**, 769–776. 10.1172/JCI118476 (1996). 8609234 10.1172/JCI118476 PMC507115

[29] Gomez, D. *et al.* Interleukin-1beta has atheroprotective effects in advanced atherosclerotic lesions of mice. *Nat. Med.* **24**, 1418–1429. 10.1038/s41591-018-0124-5 (2018). 30038218 10.1038/s41591-018-0124-5 PMC6130822

[30] Tardif, J. C. *et al.* Efficacy and safety of low-dose colchicine after myocardial infarction. *N. Engl. J. Med.* **381**, 2497–2505. 10.1056/NEJMoa1912388 (2019). 31733140 10.1056/NEJMoa1912388

[31] Taylor, E. W. The mechanism of colchicine inhibition of mitosis. I. Kinetics of inhibition and the binding of H^3^-colchicine. *J. Cell Biol.* **25**, 145–160. 10.1083/jcb.25.1.145 (1965). 10.1083/jcb.25.1.145 PMC2106604 14342828

[32] Martinon, F., Petrilli, V., Mayor, A., Tardivel, A. & Tschopp, J. Gout-associated uric acid crystals activate the NALP3 inflammasome. *Nature* **440**, 237–241. 10.1038/nature04516 (2006). 16407889 10.1038/nature04516

[33] Cecconi, A. *et al.* Effects of colchicine on atherosclerotic plaque stabilization: A multimodality imaging study in an animal model. *J. Cardiovasc. Transl. Res.* **14**, 150–160. 10.1007/s12265-020-09974-7 (2021). 32140929 10.1007/s12265-020-09974-7

[34] Helseth, R. *et al.* Tocilizumab increases citrullinated histone 3 in non-ST segment elevation myocardial infarction. *Open Heart* **8**, e001492. 10.1136/openhrt-2020-001492 (2021). 33972404 10.1136/openhrt-2020-001492 PMC8112443

[35] Huse, C. *et al.* Interleukin-6 inhibition in ST-elevation myocardial infarction: Immune cell profile in the randomised ASSAIL-MI trial. *EBioMedicine* **80**, 104013. 10.1016/j.ebiom.2022.104013 (2022). 35504178 10.1016/j.ebiom.2022.104013 PMC9079006

[36] Ridker, P. M. *et al.* Low-dose methotrexate for the prevention of atherosclerotic events. *N. Engl. J. Med.* **380**, 752–762. 10.1056/NEJMoa1809798 (2019). 30415610 10.1056/NEJMoa1809798 PMC6587584

[37] Guo, L. L. *et al.* Blocking interleukin-1 beta reduces the evolution of thoracic aortic dissection in a rodent model. *Eur. J. Vasc. Endovasc. Surg.* **60**, 916–924. 10.1016/j.ejvs.2020.08.032 (2020). 33004280 10.1016/j.ejvs.2020.08.032

[38] Jiang, Y. F. *et al.* Local upregulation of interleukin-1 beta in aortic dissecting aneurysm: Correlation with matrix metalloproteinase-2, 9 expression and biomechanical decrease. *Interact. Cardiovasc. Thorac. Surg.* **28**, 344–352. 10.1093/icvts/ivy256 (2019). 30169834 10.1093/icvts/ivy256

[39] Johnston, W. F. *et al.* Inhibition of interleukin-1beta decreases aneurysm formation and progression in a novel model of thoracic aortic aneurysms. *Circulation* **130**, S51-59. 10.1161/CIRCULATIONAHA.113.006800 (2014). 25200056 10.1161/CIRCULATIONAHA.113.006800 PMC5097450

[40] Merhi-Soussi, F. *et al.* Interleukin-1 plays a major role in vascular inflammation and atherosclerosis in male apolipoprotein E-knockout mice. *Cardiovasc. Res.* **66**, 583–593. 10.1016/j.cardiores.2005.01.008 (2005). 15914123 10.1016/j.cardiores.2005.01.008

[41] Maurice, P. *et al.* Elastin fragmentation and atherosclerosis progression: The elastokine concept. *Trends Cardiovasc. Med.* **23**, 211–221. 10.1016/j.tcm.2012.12.004 (2013). 23561795 10.1016/j.tcm.2012.12.004

[42] Sun, Q. *et al.* Elastin imaging enables noninvasive staging and treatment monitoring of kidney fibrosis. *Sci. Transl. Med.* **11**, eaat4865. 10.1126/scitranslmed.aat4865 (2019). 30944168 10.1126/scitranslmed.aat4865 PMC7115882

[43] Collettini, F. *et al.* Elastin-specific MRI of extracellular matrix-remodelling following hepatic radiofrequency-ablation in a VX2 liver tumor model. *Sci. Rep.* **11**, 6814. 10.1038/s41598-021-86417-6 (2021). 33767303 10.1038/s41598-021-86417-6 PMC7994448

[44] Coleman, R., Hayek, T., Keidar, S. & Aviram, M. A mouse model for human atherosclerosis: Long-term histopathological study of lesion development in the aortic arch of apolipoprotein E-deficient (E0) mice. *Acta Histochem.* **108**, 415–424. 10.1016/j.acthis.2006.07.002 (2006). 17007910 10.1016/j.acthis.2006.07.002

[45] Ferraz, M. L. *et al.* Correlation of lifetime progress of atherosclerosis and morphologic markers of severity in humans: New tools for a more sensitive evaluation. *Clinics (Sao Paulo)* **67**, 1071–1075. 10.6061/clinics/2012(09)15 (2012). 23018306 10.6061/clinics/2012(09)15 PMC3438249

[46] Knouff, C. *et al.* Apo E structure determines VLDL clearance and atherosclerosis risk in mice. *J. Clin. Invest.* **103**, 1579–1586. 10.1172/JCI6172 (1999). 10359567 10.1172/JCI6172 PMC408371

[47] Pendse, A. A., Arbones-Mainar, J. M., Johnson, L. A., Altenburg, M. K. & Maeda, N. Apolipoprotein E knock-out and knock-in mice: Atherosclerosis, metabolic syndrome, and beyond. *J. Lipid Res.* **50**(Suppl), S178-182. 10.1194/jlr.R800070-JLR200 (2009). 19060252 10.1194/jlr.R800070-JLR200 PMC2674752

[48] Smith, D. D. *et al.* Increased aortic atherosclerotic plaque development in female apolipoprotein E-null mice is associated with elevated thromboxane A2 and decreased prostacyclin production. *J. Physiol. Pharmacol.* **61**, 309–316 (2010). 20610861 PMC3515053

[49] Centner, A. *et al.* Sex differences in atherosclerosis in ApoE^-/-^ mice exposed to nicotine and cigarette smoke. *Faseb J.*10.1096/fasebj.2021.35.S1.04018 (2021). 10.1096/fasebj.2021.35.S1.04018

[50] Isoda, K. *et al.* Inhibition of interleukin-1 suppresses angiotensin II-induced aortic inflammation and aneurysm formation. *Int. J. Cardiol.* **270**, 221–227. 10.1016/j.ijcard.2018.05.072 (2018). 29884291 10.1016/j.ijcard.2018.05.072

[51] Awan, Z. *et al.* Reducing vascular calcification by anti-IL-1β monoclonal antibody in a mouse model of familial hypercholesterolemia. *Angiology* **67**, 157–167. 10.1177/0003319715583205 (2016). 25904765 10.1177/0003319715583205

[52] Geiger, T. *et al.* Neutralization of interleukin-1 beta activity in vivo with a monoclonal antibody alleviates collagen-induced arthritis in DBA/1 mice and prevents the associated acute-phase response. *Clin. Exp. Rheumatol.* **11**, 515–522 (1993). 8275587

[53] Reimann, C. *et al.* Dual-probe molecular MRI for the in vivo characterization of atherosclerosis in a mouse model: Simultaneous assessment of plaque inflammation and extracellular-matrix remodeling. *Sci. Rep.* **9**, 13827. 10.1038/s41598-019-50100-8 (2019). 31554825 10.1038/s41598-019-50100-8 PMC6761132

[54] Brangsch, J. *et al.* Concurrent molecular magnetic resonance imaging of inflammatory activity and extracellular matrix degradation for the prediction of aneurysm rupture. *Circ. Cardiovasc. Imaging* **12**, e008707. 10.1161/CIRCIMAGING.118.008707 (2019). 30871334 10.1161/CIRCIMAGING.118.008707

[55] Adams, L. C. *et al.* Simultaneous molecular MRI of extracellular matrix collagen and inflammatory activity to predict abdominal aortic aneurysm rupture. *Sci. Rep.* **10**, 15206. 10.1038/s41598-020-71817-x (2020). 32939002 10.1038/s41598-020-71817-x PMC7494914

[56] Adams, L. C. *et al.* Noninvasive imaging of vascular permeability to predict the risk of rupture in abdominal aortic aneurysms using an albumin-binding probe. *Sci. Rep.* **10**, 3231. 10.1038/s41598-020-59842-2 (2020). 32094414 10.1038/s41598-020-59842-2 PMC7039902

[57] Botnar, R. M. *et al.* In vivo assessment of aortic aneurysm wall integrity using elastin-specific molecular magnetic resonance imaging. *Circ. Cardiovasc. Imaging* **7**, 679–689. 10.1161/CIRCIMAGING.113.001131 (2014). 24871347 10.1161/CIRCIMAGING.113.001131

